# Evaluation of strategies for identification of infants with pathogenic glucose-6-phosphate dehydrogenase variants in China

**DOI:** 10.3389/fgene.2022.844381

**Published:** 2022-09-23

**Authors:** Zhongmin Xia, Xudong Wang, Huiming Ye, Chunliu Gao, Xiaoman Zhou, Jing Chen, Yunsheng Ge, Juan Li, Yulin Zhou, Qiwei Guo

**Affiliations:** ^1^ United Diagnostic and Research Center for Clinical Genetics, Women and Children’s Hospital, School of Medicine and School of Public Health, Xiamen University, Xiamen, Fujian, China; ^2^ Department of Clinical Laboratory, Women and Children’s Hospital, School of Medicine, Xiamen University, Xiamen, Fujian, China; ^3^ Department of Child Health, Women and Children’s Hospital, School of Medicine, Xiamen University, Xiamen, Fujian, China

**Keywords:** G6PD deficiency, enzymatic activity, genotyping, cost analysis, diagnostic performance

## Abstract

Glucose-6-phosphate dehydrogenase (G6PD) deficiency, which is caused by pathogenic variants of *G6PD* that result in decreased G6PD activity, is an X-linked inherited inborn error of metabolism that occurs worldwide. Individuals with G6PD deficiency and heterozygous females with normal G6PD activity (i.e., all individuals with pathogenic *G6PD* variants) are at risk of developing hemolytic anemia under increased oxidative challenge. However, this risk can be minimized by timely diagnosis. Currently, two assays are used to diagnose G6PD deficiency in China: evaluation of enzymatic activity and targeted genotyping. In terms of identification of all individuals with pathogenic *G6PD* variants, the performance and cost of different diagnostic strategies (isolated or combined evaluation of G6PD activity and *G6PD* genotyping) can vary, and these factors should be comprehensively evaluated. In this study, we examined 555 infants (437 males and 118 females) who were positive for the newborn screening of G6PD deficiency. We first evaluated the diagnostic performances of enzymatic testing and targeted genotyping. Both assays attained 100% specificities and positive predictive values for both male and female infants. In contrast, the sensitivities and negative predictive values (NPVs) of the diagnostic tests were different for male and female infants. For male infants, the sensitivities were 99.8 and 98.3%, and the NPVs were 94.1% and 69.6%, for enzymatic testing and targeted genotyping, respectively. For female infants, the sensitivities were 62.5% and 97.9%, and the NPVs were 37.9% and 91.7%, for enzymatic testing and targeted genotyping, respectively. We also evaluated the cost of the five different diagnostic strategies. The combination of G6PD activity testing of all infants, followed by genotyping of female infants with normal G6PD activity, attained high diagnostic sensitivity (99.8%) at a low cost (8.60 USD per diagnosed case). In the future, simultaneous examination of G6PD activity and whole-exon or whole-gene *G6PD* sequencing could become a standard clinical practice. Our data provide references for clinical practice on the standardization of current and future interventions for G6PD deficiency in China.

## Introduction

Glucose-6-phosphate dehydrogenase (G6PD), which is ubiquitously expressed in mammalian cells, functions as an oxidoreductase that catalyzes the oxidation of glucose-6-phosphate to 6-phosphogluconolactone while reducing nicotinamide adenine dinucleotide phosphate (NADP) to NADPH (Stanton 2012; Luzzatto, et al., 2020). NADPH is an electron donor that plays key roles in numerous biological processes, such as the biosynthesis of deoxyribonucleotides and fatty acids, metabolism of many drugs and xenobiotics, and defense against oxidative challenge (Stanton 2012; Luzzatto, et al., 2020). NADPH is an important antioxidant in red blood cells as it is involved in the enzymatic pathways that remove intracellular H_2_O_2_ (Stanton 2012; Luzzatto, et al., 2020). G6PD deficiency, caused by pathogenic variants of *G6PD* that result in decreased G6PD activity, is an X-linked inherited inborn error of metabolism that was the first identified red cell enzymopathy (Alving, et al., 1956). It is estimated that over 500 million people are affected by G6PD deficiency worldwide (Nkhoma, et al., 2009; Luzzatto, et al., 2020). However, the prevalence varies among different populations and is relatively high in Africa, Southern Europe, the Middle East, Southeast Asia, and the Mediterranean (Nkhoma, et al., 2009; Howes, et al., 2012). In China, the prevalence of G6PD deficiency varied in different provinces with regional and ethnic features and the integrated prevalence is approximately 0.767% (Liu, et al., 2020).

Although most individuals with G6PD deficiency are asymptomatic throughout their lifetime, they are at risk of developing acute, sometimes fatal, hemolytic anemia when oxidative challenge increases in red blood cells (Luzzatto, et al., 2020). Such scenarios can occur following the ingestion of fava beans, the intake of specific drugs (e.g., primaquine and rasburicase), and, in rare cases, infection (Luzzatto and Arese 2018; Luzzatto, et al., 2020; Onori, et al., 2021; Yang, et al., 2021). However, the risk of hemolytic anemia can be minimized by timely diagnosis of G6PD deficiency because the intake of these foods and drugs can then be avoided. In China, newborn screening (NBS) for G6PD deficiency, in which a fluorescent spot test is used to evaluate G6PD activity in a dried blood spot sample (Wang, et al., 2021), is the first-tier test for the timely diagnosis of G6PD deficiency. As illustrated in our previous study, infants with positive screening results, who are considered at high risk for G6PD deficiency, are recalled and subjected to further diagnostic testing to identify the G6PD-deficient infants (Wang, et al., 2021). In addition to G6PD-deficient infants, female infants with heterozygous pathogenic *G6PD* variants whose G6PD activity levels are normal should also be identified. These females are at risk of G6PD deficiency-associated complications, such as hemolysis when challenged by oxidizing agents (Shah, et al., 2012). In this sense, all infants with pathogenic *G6PD* variants should be diagnosed.

Several assays have been developed for the diagnosis of G6PD deficiency worldwide (Alam, et al., 2018; Ley, et al., 2022; Pal, et al., 2019; Roh, et al., 2016; von Fricken, et al., 2014; Zobrist, et al., 2021). Currently, two assays are approved by the domestic Food and Drug Administration for use in diagnosis of G6PD deficiency in China: evaluation of G6PD activity and targeted *G6PD* genotyping (Liu, et al., 2020; Luzzatto, et al., 2020). These two assays have been used in the national NBS program which delineated the profiles of G6PD deficiency in China (Liu, et al., 2020). Compared with G6PD activity testing, targeted genotyping has better sensitivity for diagnosing female heterozygotes (Xia, et al., 2016; Islam, et al., 2018). Previously, we reported the NBS program in Xiamen City and indicated a 1.39% prevalence of G6PD deficiency in this city (Wang, et al., 2021). Moreover, we noticed that in clinical practice of China, the diagnostic strategy (isolated or combined evaluation of G6PD activity and *G6PD* genotyping) varies and mainly depends on the opinions of physicians or the choice of guardians after genetic counseling. In terms of identification of all infants with pathogenic *G6PD* variants, the performance of different diagnostic strategies could differ and should be comprehensively evaluated, which is urgent considering the amount of affected babies in China. Moreover, in an era of globally contracting health care resources, the costs of diagnostic strategies are crucial concerns, especially in underdeveloped areas with a high prevalence of this genetic defect.

In this study, we aimed to evaluate the performances and costs of diagnostic strategies that are approved and frequently used in China for diagnosing individuals with pathogenic *G6PD* variants from infants with positive NBS results.

## Materials and methods

### Subjects

Based on NBS and diagnostic procedures in China (Wang, et al., 2021), infants with positive NBS results were recalled for diagnostic testing for G6PD deficiency. After obtaining informed consent from guardians, 555 infants (437 males and 118 females) with positive NBS results were recruited in 2020 in NBS center, Women and Children’s Hospital of Xiamen University. These infants underwent diagnostic G6PD activity testing and targeted *G6PD* variant genotyping simultaneously. The ages of the infants at the time of sampling are shown in Table 1.

**TABLE 1:** Age of infants at the time of sampling for the diagnostic testing of G6PD deficiency.

Age (day)	Number of males	Number of females	Total number
1–30	240	49	289^a^
31–60	155	54	209
61–90	20	8	28
91–120	8	3	11
121–150	7	4	11
151–180	7	0	7
Total	437	118	555

a In Chinese tradition, people believe that keeping puerpera and neonates at home for approximately 1 month after labor would be good for their health. Although receiving the recall information before 2 weeks after labor, a number of families did not bring their neonates for diagnosis immediately if the neonates did not have visible clinical phenotypes. Therefore, only 289 of 555 neonates get the confirmatory test before 1 month of life.

### Diagnostic testing of G6PD activity

G6PD activity was evaluated by determining the ratio of G6PD to 6-phosphogluconate dehydrogenase (6PGD) using the nitroblue tetrazolium G6PD/6PGD test kit (approved number: YZB/0829–2011, Micky Med, Guangzhou, Guangdong, China) according to the manufacturer’s protocol. Briefly, 50 uL of EDTA-anticoagulated peripheral blood was used to evaluate the G6PD activity based on the quantification of the product of enzymatic reaction (i.e., NAPDH). In the meantime, 6PGD activity was evaluated as an internal control. A newborn with a G6PD/6PGD ratio <1.0 was considered G6PD-deficient, whereas a G6PD/6PGD ratio ≥1.0 was considered G6PD-normal (Chen, et al., 2018; Fu, et al., 2018; Liu, et al., 2020).

### Diagnostic genotyping of target *G6PD* variants

Genomic DNA was extracted from 100 to 200 μl of EDTA-anticoagulated peripheral blood with the Super/HF16 plus DNA Extraction System (MagCore, Xiamen, China). After quantification with a Nanodrop 2000TM Spectrophotometer (Thermo Fisher Scientific, Wilmington, DE, United States), 10 ng of extracted DNA was analyzed using the MeltPro^®^ *G6PD* genotyping kit (approved number: 20153400623, Zeesan Biotech, Xiamen, Fujian, China) to detect 12 common *G6PD* variants: c.95A > G (Gaohe), c.383T > C (Vanua Lava), c.392G > T (Quing Yan), c.487G > A (Mahidol), c.517T > C (Nankang), c.592C > T (Coimbra), c.871G > A (Viangchan), c.1004C > A (Fushan), c.1024C > T (Chinese-5), c.1360C > T (Maewo), c.1376G > T (Canton), and c.1388G > A (Kaiping). The reactions were performed on a SLAN-96 real-time PCR system (Hongshi, Shanghai, China). The PCR amplification and melting curve analyses have been described in detail in a previous report (Wang, et al., 2021).

### Sanger sequencing of *G6PD* exons

Sanger sequencing was considered as the gold standard test to evaluate the performance of diagnostic tests. However, since the targeted genotyping demonstrated 100% concordance with Sanger sequencing for all targeted variants (Xia, et al., 2016), we only sequenced those samples which were negative for targeted genotyping instead of sequencing all samples. To perform Sanger sequencing of the *G6PD* coding exons (exons 2–13), genomic DNA (10 ng) was amplified with sequencing primers (Supplementary Table S1) on an ABI-Verity Thermal Cycler (Applied Biosystems, Foster City, CA, United States). The PCR conditions are described in detail in Supplementary Table S2. After amplification, the PCR products were analyzed by a commercial sequencing service (Sangon, Shanghai, China).

### Cost analysis

The self-pay costs of these tests were calculated based on the median charges in three provinces with different levels of economic development (Fujian, Guangdong, and Jiangxi) (G6PD activity evaluation: $3; targeted genotyping: $50). The cost of diagnostic testing is denoted as the sum of the cost for diagnostic testing in a specific strategy branch, while the cost of testing per diagnosed case is denoted as the average cost for each diagnosed G6PD deficiency case in a specific strategy branch. The costs of all tests are expressed in United States dollars (USD), with a hypothetical exchange rate of 6.8 to the Chinese currency.

### Ethics statement

Signed informed consent was obtained from the guardians of all study subjects. The study protocol was approved by the research ethics committees of the Women and Children’s Hospital at the School of Medicine of Xiamen University.

## Results

### Diagnostic performances of enzymatic testing and targeted genotyping in male infants

**FIGURE 1:**
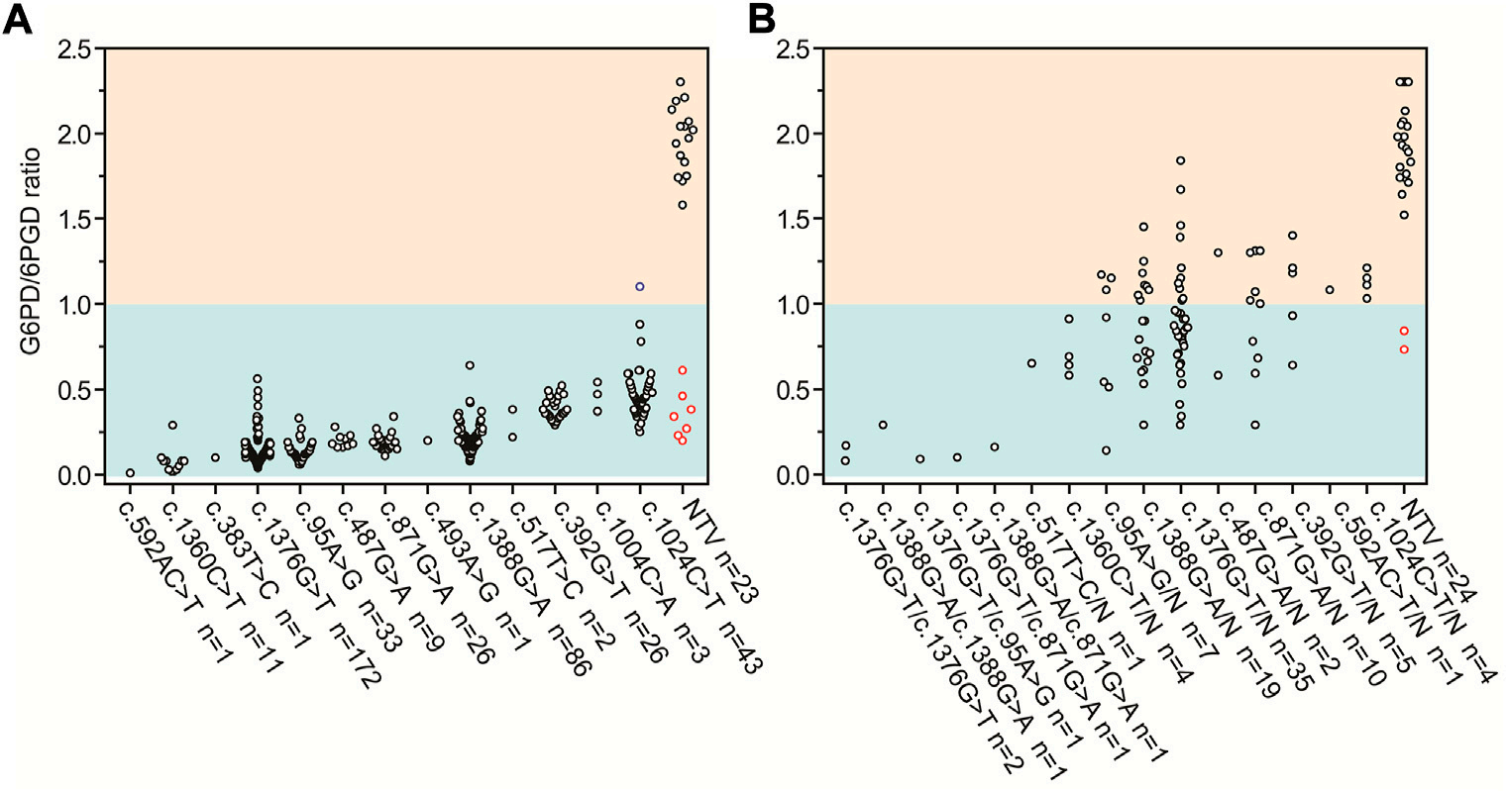
G6PD activities and targeted *G6PD* genotypes for **(A)** 437 male and **(B)** 118 female infants with positive newborn screening results. Red circles denote infants with G6PD activity deficiencies but negative for any of the target *G6PD* variants. Blue circle denotes the infant with the c.1024C > T variant and normal G6PD activity. G6PD, glucose-6-phosphate dehydrogenase; 6PGD, 6-phosphogluconate dehydrogenase; n, number; N, normal allele; NTV, negative for target variants.

The G6PD activity levels and *G6PD* variants of 437 tested male infants are shown in Figure 1A 420 infants were diagnosed with G6PD deficiency and 414 infants carried pathogenic variants based on the results of the G6PD activity test and targeted genotyping, respectively. Seven infants with G6PD activity deficiencies did not carry any of the target variants, and one infant with the c.1024C > T variant had normal G6PD activity (Figure 1A). 23 infants negative for targeted genotyping were examined using Sanger sequencing. The results showed that seven G6PD-defecient infants carried *G6PD* variants outside the detection regions of the MeltPro^®^ *G6PD* genotyping kit (Figure 1A; Table 2), and the c.1024C > T variant was confirmed for the case with normal G6PD activity (Figure 1A). Because the c.1024C > T variant is a known pathogenic variant, this case was considered as a G6PD deficiency. Therefore, 421 out of 437 male infants carried pathogenic *G6PD* variants. The diagnostic performances of G6PD activity testing and targeted genotyping were listed in Table 3.

**TABLE 2:** Sanger sequencing results of G6PD deficient cases who were negative for targeted genotyping.

Case number	Sex	Age	G6PD/6PGD	Variant
1	Male	1 m 8 d	0.61	c.98T > C
2	Male	1 m 4 d	0.34	c.202G > A (Asahi)
3	Male	19 d	0.27	c.305T > C
4	Male	1 m 9 d	0.23	c.305T > C
5	Male	29 d	0.20	c.305T > C
6	Male	1 m 11 d	0.46	c.739G > A
7	Male	2 m 16 d	0.38	c.835A > T (Chinese-1)
8	Female	1 m	0.84	Heterozygous c.305T > C
9	Female	26 d	0.73	Heterozygous c.563C > T (Mediterranean)

m, month; d, day.

**TABLE 3:** Diagnostic performances of enzymatic testing and targeted genotyping.

	NBS-positive male infants		NBS-positive female infants	
	Enzymatic testing	Targeted genotyping	Enzymatic testing	Targeted genotyping
Sensitivity	99.8% (420/421)	98.3% (414/421)	62.5% (60/96)	97.9% (94/96)
Specificity	100% (16/16)	100% (16/16)	100% (22/22)	100% (22/22)
Positive predictive value	100% (420/420)	100% (414/414)	100% (60/60)	100% (94/94)
Negative predictive value	94.1% (16/17)	69.6% (16/23)	37.9% (22/58)	91.7% (22/24)

NBS, newborn screening.

### Diagnostic performances of enzymatic testing and targeted genotyping in female infants

The G6PD activity levels and *G6PD* variants of 118 female infants are shown in Figure 1B. 60 infants were diagnosed with G6PD deficiency and 94 infants carried pathogenic variants based on the results of the G6PD activity test and targeted genotyping, respectively. Two infants with G6PD activity deficiencies did not carry target variants, and 36 heterozygous infants with target variants had normal G6PD activity levels (Figure 1B). 24 infants negative for targeted genotyping were examined using Sanger sequencing. The results showed that two G6PD-deficient cases carry *G6PD* variants outside the detection range of the MeltPro^®^ *G6PD* genotyping kit (Table 2). Therefore, 96 out of 118 female infants carried pathogenic *G6PD* variants. In terms of identification of these cases, the diagnostic performances of G6PD activity testing and targeted genotyping were listed in Table 3.

### Comparison of different strategies for the diagnosis of G6PD deficiency

**FIGURE 2:**
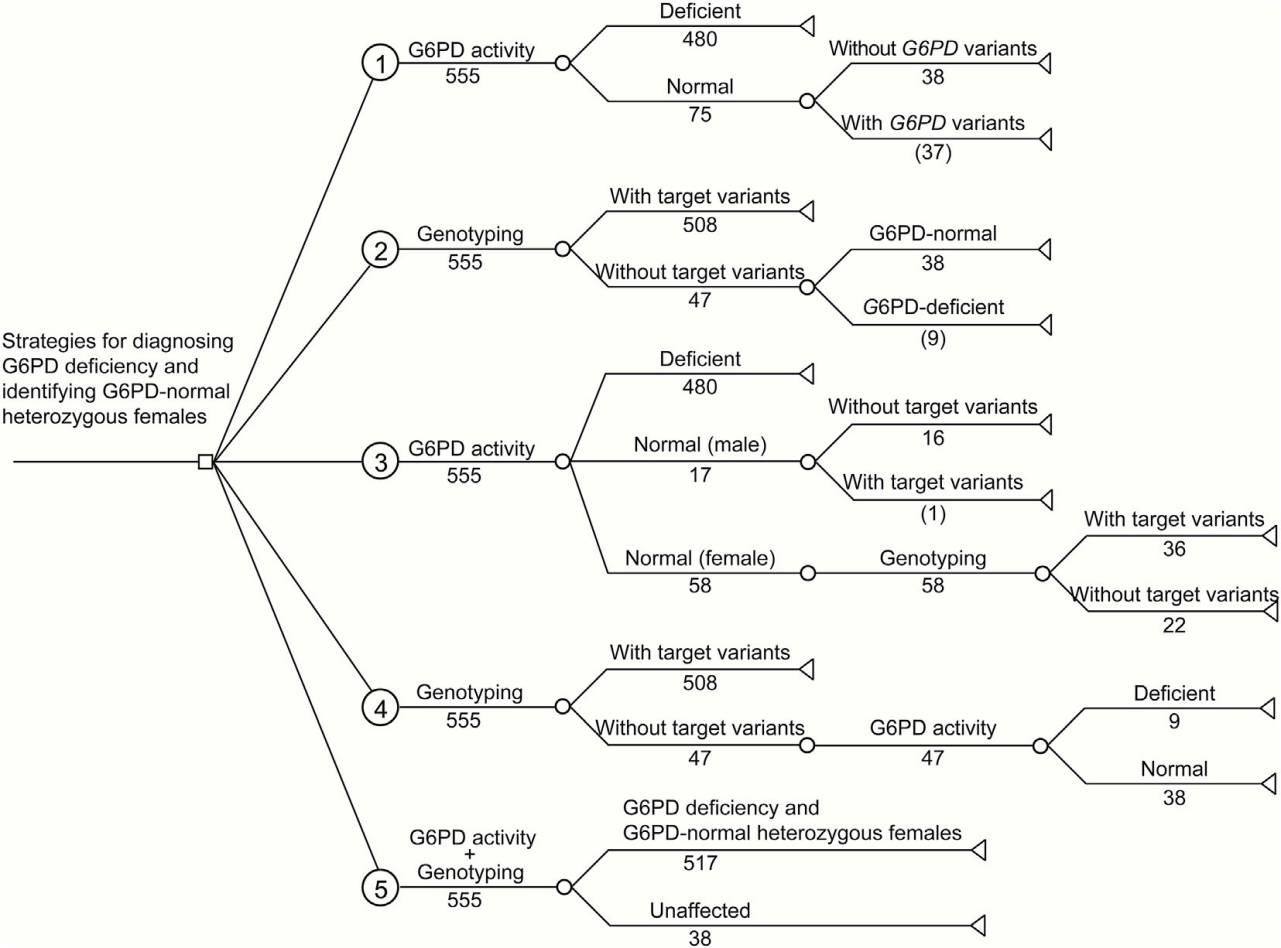
Decision-analytic model of five strategies for diagnosing G6PD deficiency and identifying G6PD-normal heterozygous females from infants with positive newborn screening results. Based on the experimental results, the numbers of cases are listed. Number in parenthesis indicates the number of infants with missing diagnosis or identification.

Based on the G6PD activities and genotypes of the 555 analyzed infants, a simplified decision-analytic model was developed to compare the cost of five diagnostic strategies for G6PD deficiency (Figure 2). In strategies 1 and 2, G6PD activity and target *G6PD* variants were independently evaluated, respectively. In strategy 3, all infants were evaluated for G6PD activity, and then female infants with normal G6PD activity levels were examined for target variants. In strategy 4, all infants were first evaluated for target variants, and then G6PD activity levels were examined in infants without target variants. In strategy 5, G6PD activity levels and target variants were simultaneously evaluated for all infants. The cost analysis of different strategies is listed in Table 4.

**TABLE 4:** Cost analysis of different strategies for the diagnosis of G6PD deficiency and female heterozygotes from infants with positive newborn screening results.

Strategy	Diagnosed/undiagnosed cases	Diagnostic sensitivity (%)	Information of G6PD activity	Information of genotype	Genetic information		Cost of diagnostic testing	Cost of testing per diagnosed case
					Allele frequency	Genotype-phenotype correlation		
1	480/37	92.8	555	0			1,665	3.47
2	508/9	98.3	0	555	√		27,750	54.63
3	516/1	99.8	555	58			4,623	8.60
4	517/0	100	47	555	√		27,891	53.95
5	517/0	100	555	555	√	√	29,415	56.90

## Discussion

G6PD deficiency is typically referred to as an X-linked recessive genetic trait. However, this is inaccurate because heterozygous females can also be affected (Luzzatto, et al., 2020). The ratio of normal to deficient red blood cells is highly variable among heterozygous females, and their phenotypic spectrum is wide, ranging from normal to G6PD deficiency, like that of hemizygous males (Figure 1B) (He et al., 2020; Wang et al., 2021). Moreover, even in an individual heterozygous female, G6PD activity may vary under different physiological or pathological conditions (Au et al., 2006). Therefore, heterozygous females, regardless of whether their G6PD activity levels are normal in a single assay, should be considered at risk of G6PD deficiency-associated complications, such as hemolysis when challenged by oxidizing agents. In this sense, heterozygous females require a definite diagnosis as much as hemizygous males (Shah et al., 2012).

To identify all infants with pathogenic *G6PD* variants, the enzymatic testing and targeted genotyping attained 100% specificities and positive predictive values for both male and female infants. In contrast, the sensitivities and negative predictive values (NPVs) of the diagnostic tests were different for male and female infants. For male infants, diagnostic sensitivity and NPV were higher for G6PD activity-based methods than for targeted genotyping (Table 3). Cases with G6PD activity deficiency but no target variants carried pathogenic variants outside the detection range (Table 2), confirming that all cases with deficient G6PD activity had genetic causes. In this sense, the diagnostic sensitivities and NPVs of the two methods would be equivalent if the detection range of genotyping was expanded. Notably, one infant with the c.1024C > T variant had normal G6PD activity according to the cut-off value of the detection kit. However, the G6PD activities of infants with the c.1024C > T variant were distinct from those of infants who did not carry variants (Figure 1A). Therefore, we attribute this discordance to the imperfect cut-off value, suggesting that further evaluation and optimization of the cut-off value be considered by the kit manufacturer. We concluded that for the diagnosis of G6PD deficiency in male infants, evaluating G6PD activity had better performance than targeted genotyping. However, if the detection range of genotyping were to be expanded, these methods could have equivalent performance.

In contrast to what was observed for male infants, the diagnostic sensitivity and NPV were noticealy lower for the G6PD activity-based method than for targeted genotyping in female infants (Table 3). This is because a considerable portion of heterozygous females with normal G6PD activity was missed by the enzymatic testing. Therefore, for the diagnosis of all heterozygotes in female infants, targeted genotyping outperformed G6PD activity testing. Moreover, similar to the findings in male infants, the diagnostic sensitivities of genotyping will increase with the expansion of detected genotypes, as two additional variants were found by Sanger sequencing (Table 2). It should, however, be noted that the G6PD activity test is still very useful for female heterozygotes because it is most predictive of the potential severity of hemolysis.

As shown in Table 4, independent testing of G6PD activity (strategy 1) was the most economical strategy in terms of the cost spent on a diagnosed case (3.47 USD), but had the lowest diagnostic sensitivity (92.8%). In comparison, due to the higher diagnostic sensitivity of genotyping for female infants, the use of genotyping, either independently (strategy 2) or combined with G6PD activity testing (strategies 3–5), noticeably increased the diagnostic sensitivity to 98.3%–100%. Moreover, genotyping enables us to study the genetic characteristics of G6PD deficiency in a specific population (Figure 1), such as allele frequency (strategies 2, 4, and 5) and genotype–phenotype correlations (strategy 5), which are valuable for our understanding of such genetic defects (Liu et al., 2020; Wang et al., 2021). However, in the current state, genotyping markedly increases the testing cost spent on a diagnosed case (Table 4). Therefore, the preferred diagnostic strategy depends on the objective and expectations. For example, for diagnosis only, strategy 3 is recommended. In this strategy, inexpensive testing of G6PD activity is used as a first-tier diagnostic method for all infants, and then genotyping is applied for female infants with normal G6PD activity to further identify heterozygous female infants. This strategy attained high diagnostic sensitivity (99.8%) but retained relatively low testing cost (8.60 USD per diagnosed case) compared to other strategies with comparable diagnostic sensitivities (strategies 4 and 5). Therefore, strategy 3 would be more favorable in underdeveloped regions where medical cost is a crucial concern. It should be noted that the methodology, accuracy, detection range, and the cost of enzymatic activity analysis and genotyping may vary in different countries and regions, suggesting re-evaluation of cost-effectiveness using this decision-analytical model on a case-by-case basis. Moreover, the cost of strategy 3 changes with the proportion of female infants, as more genotyping tests would be needed when the proportion of female infants increases. However, genotyping of *G6PD* is essential not only for diagnosis but also for epidemiological investigations and genotype-phenotype correlation studies, which are of great significance for genetic counseling, prognosis prediction, and the management of G6PD deficiency. Therefore, for the purpose of attaining comprehensive genetic information, simultaneous evaluation of G6PD activity and *G6PD* variants (strategy 5) is recommended despite the considerable cost of genotyping, as it is the only strategy to evaluate genotype-phenotype correlations (Table 4). Notably, with the development of detection methodologies, the detection range and cost of genotyping are continuously increasing and decreasing, respectively. For example, high-throughput sequencing, in which hundreds of genes, including *G6PD*, can be simultaneously and comprehensively examined, has very recently been used for expanded newborn screening (Luo, et al., 2020; Hao, et al., 2021). In this context, the cost for each target gene is limited. Therefore, it is reasonable to expect that simultaneous examination of G6PD activity and sequencing of the entire *G6PD* gene or exons will become the standard clinical practice for the diagnosis of G6PD deficiency in the near future.

In conclusion, we demonstrated the diagnostic performance of testing based on G6PD activity and targeted *G6PD* genotyping and evaluated the performance and cost of different strategies for the diagnosis of infants with pathogenic *G6PD* variants. Currently, testing all infants for G6PD activity, followed by genotyping of female infants with normal G6PD activity could attain high diagnostic sensitivity at a low cost. In the future, simultaneous examination of G6PD activity and sequencing of the entire *G6PD* gene or exons could become standard practice for the diagnosis of G6PD deficiency and those at risk of G6PD deficiency-associated complications. Our data provide references for the standardization of current and future interventions for G6PD deficiency.

## Data Availability

The original contributions presented in the study are included in the article/supplementary materials, further inquiries can be directed to the corresponding authors.
