# Supplementary material for: Evaluation of strategies for identification of infants with pathogenic glucose-6-phosphate dehydrogenase variants in China
Source: Front Genet. 2022 Sep 23;13:844381. doi: 10.3389/fgene.2022.844381 (PMC9538342; doi:10.3389/fgene.2022.844381)
Supplement: Supplementary file 1 [file DataSheet1.docx]

**Supplemental Table 1. The list of sequencing primers.**

| **Name** | **Sequence (5’-3’)** |
| --- | --- |
| F1 | GTGATTTGGGCAATCAGGTGTC |
| R1 | GATGATCCTGGCGCACTAGC |
| F2 | TGATGGCACCTCAGAGTTACC |
| R2 | CACAGCAAGAATCTTATCAGACCAAT |
| F3 | CCAGTAGTGATCCTGAGTAGTGC |
| R3 | CAGGAGAGGAGGAGAGCATC |
| F4 | GGGACGGGGACACTGACTT |
| R4 | CCCCGGACACGCTCATAGA |
| F5 | GGTCATCTGGGAACACAAGGC |
| R5 | GAGGAGCTCCCCCAAGATAGG |
| F6 | GCCCTTGAACCAGGTGAACA |
| R6 | GGTGACTTCTCCGGGGTTGA |
| F7 | AGGGTGAGCAGAGCCAAGC |
| R7 | GCTCGTCTGCCTCCGTGG |

F: forward primer; R: reverse primer.

**Supplemental Table 2. PCR cycling condition for sanger sequencing.**

|  | **Conditions** | **Cycle number** |
| --- | --- | --- |
| PCR amplification | 95 °C, 10 min | 1 |
|  | 95 °C, 30 s | 40 |
|  | 63 °C, 30 s |  |
|  | 76 °C, 45 s |  |
|  | 76 °C, 10 min | 1 |
